# MRI-based radiomics nomogram for differentiation of solitary metastasis and solitary primary tumor in the spine

**DOI:** 10.1186/s12880-023-00978-8

**Published:** 2023-02-09

**Authors:** Sha Li, Xinxin Yu, Rongchao Shi, Baosen Zhu, Ran Zhang, Bing Kang, Fangyuan Liu, Shuai Zhang, Ximing Wang

**Affiliations:** 1 Shandong Provincial Hospital, Shandong University, No. 44, Wenhua West Road, Jinan, 250012 Shandong China; 2 Huiying Medical Technology Co. Ltd, Beijing, China; 3 School of Medicine, Shandong First Medical University, No. 6699, Qingdao Road, Jinan, 250024 Shandong China; 4 Department of Radiology, Shandong Provincial Hospital Affiliated to Shandong First Medical University, Shandong University, No. 324, Jingwu Road, Jinan, 250021 Shandong China

**Keywords:** Spinal tumor, Solitary spinal metastasis, Nomogram, Radiomics, Magnetic resonance imaging

## Abstract

**Background:**

Differentiating between solitary spinal metastasis (SSM) and solitary primary spinal tumor (SPST) is essential for treatment decisions and prognosis. The aim of this study was to develop and validate an MRI-based radiomics nomogram for discriminating SSM from SPST.

**Methods:**

One hundred and thirty-five patients with solitary spinal tumors were retrospectively studied and the data set was divided into two groups: a training set (n = 98) and a validation set (n = 37). Demographics and MRI characteristic features were evaluated to build a clinical factors model. Radiomics features were extracted from sagittal T1-weighted and fat-saturated T2-weighted images, and a radiomics signature model was constructed. A radiomics nomogram was established by combining radiomics features and significant clinical factors. The diagnostic performance of the three models was evaluated using receiver operator characteristic (ROC) curves on the training and validation sets. The Hosmer–Lemeshow test was performed to assess the calibration capability of radiomics nomogram, and we used decision curve analysis (DCA) to estimate the clinical usefulness.

**Results:**

The age, signal, and boundaries were used to construct the clinical factors model. Twenty-six features from MR images were used to build the radiomics signature. The radiomics nomogram achieved good performance for differentiating SSM from SPST with an area under the curve (AUC) of 0.980 in the training set and an AUC of 0.924 in the validation set. The Hosmer–Lemeshow test and decision curve analysis demonstrated the radiomics nomogram outperformed the clinical factors model.

**Conclusions:**

A radiomics nomogram as a noninvasive diagnostic method, which combines radiomics features and clinical factors, is helpful in distinguishing between SSM and SPST.

**Supplementary Information:**

The online version contains supplementary material available at 10.1186/s12880-023-00978-8.

## Introduction

The spine is a common site for metastatic disease and primary tumors, with metastatic disease being much more frequent [1]. Nearly 70% of patients with cancers develop bone metastases, and approximately 40% of cancer patients occur spinal metastases during the course of their disease [2]. Typically, metastases appear as multifocal lesions in the axial skeleton, which can be suggestive for diagnosis. However, when metastasis presents as a solitary lesion, imaging features of solitary spinal metastasis (SSM) and solitary primary spinal tumor (SPST) frequently overlap, making it difficult to distinguish by traditional imaging practice [3, 4]. In addition, although tumor history is suggestive for the diagnosis of metastasis, its help is limited [5, 6]. The treatment decisions and prognosis are noticeably different depending on the diagnosis of spinal disease [7, 8]. Therefore, differentiating SSM from SPST is essential for the prognostic evaluation of the patients and the selection of appropriate treatment.

Several studies [9, 10] presented that positron emission tomography/computed tomography (PET/CT) could provide both functional and anatomic information, and is an excellent functional imaging method to identify spinal metastasis. However, this examination may not be widely available for clinical use due to its expensive price [11]. In addition, most investigators showed that percutaneous biopsy is a precise diagnostic method for spinal disease, however, it could result in complications such as hematoma, infection, and organ damage, so not all patients can undergo biopsy [5, 12]. Therefore, an efficient and non-invasive approach to discriminate SSM from SPST is urgent.

Recently, radiomics, as an emerging method of medical image analysis, can extract and analyze quantitative features from radiographic images [13, 14]. And radiomics has been shown to be helpful in tumor detection, diagnosis, prognostic assessment, prediction of treatment response, and monitoring of disease status [15]. Sun et al. [16] demonstrated that radiomics can differentiate between benign and malignant spinal tumors. In addition, previous studies have also assessed the ability of radiomics to differentiate between spinal metastases and other pathological spinal lesions [17–19]. However, to the best of our knowledge, there have been few studies that established MRI-based radiomics nomograms to differentiate SSM and SPST.

The aim of this study was to develop and validate a radiomics nomogram incorporating radiomics signature and clinical factors for discriminating between SSM and SPST.

## Materials and methods

### Patients

Our hospital’s Institutional Review Board approved this retrospective study and the requirement for the patients’ informed consent was waived.

**Fig. 1:**
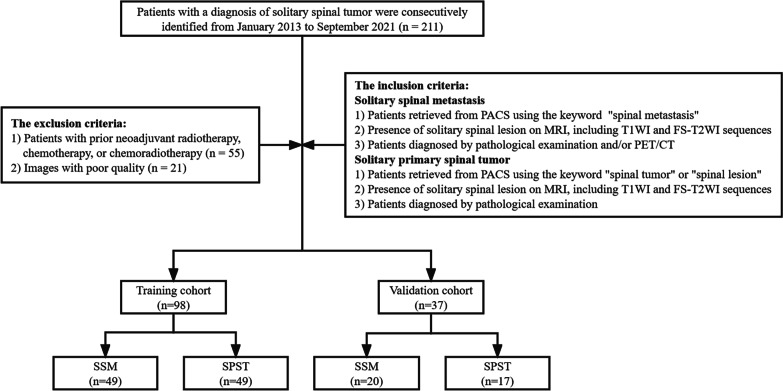
The flowchart of inclusion and exclusion criteria. *SSM* solitary spinal metastasis, *SPST* solitary primary spinal tumor

From January 2013 to September 2021, this study enrolled patients with solitary spinal tumors from our hospital. The primary cancers of SSM included lung cancer (n = 29), renal cancer (n = 14), breast cancer (n = 7), rectal cancer (n = 5), prostate cancer (n = 2), stomach cancer (n = 2), thyroid cancer (n = 2), liver cancer (n = 1), esophageal cancer (n = 1), colon cancer (n = 1), pancreatic cancer (n = 1), cervical cancer (n = 1), bladder cancer (n = 1), ovarian cancer (n = 1), submandibular gland cystadenocarcinoma (n = 1). The SPST included plasmacytoma of bone (n = 12), giant cell tumor of bone (n = 11), chordoma (n = 10), lymphoma (n = 6), hemangioma (n = 5), osteoblastoma (n = 4), langerhans cell histiocytosis (n = 4), chondrosarcoma (n = 4), ewing sarcoma (n = 2), chondromyxoid fibroma (n = 2), liposarcoma (n = 2), aneurysmal bone cyst (n = 1), osteosarcoma (n = 1), epithelioid haemangioendothelioma (n = 1), fibrosarcoma (n = 1). The inclusion criteria for SSM were as follows: (1) Patients retrieved from Picture Archiving and Communication System (PACS) using the keywords "spinal metastasis"; (2) Presence of solitary spinal lesion on MRI, including T1-weighted imaging (T1WI) and fat-suppressed T2-weighted imaging (FS-T2WI) sequences; (3) Patients confirmed by pathological examination and/or PET/CT and clinically diagnosed. The inclusion criteria for SPST were as follows: (1) Patients retrieved from PACS using the keywords "spinal tumor" or "spinal lesion"; (2) Presence of solitary spinal lesion on MRI, including T1-weighted imaging (T1WI) and fat-suppressed T2-weighted imaging (FS-T2WI) sequences; (3) Patients diagnosed by pathological examination. The common exclusion criteria were as follows: (1) Patients with neoadjuvant radiotherapy, chemotherapy, or chemoradiotherapy before MRI; (2) Images with poor quality, such as motion artifacts, cardiac and large vessel pulsation artifacts. All patients were randomized (a 7:3 ratio) into training and validation cohorts. The flowchart of inclusion and exclusion criteria for this study is shown in Fig. 1.

### MRI protocols

The MRI was performed on 3.0 Tesla MR scanner (Prisma, Siemens Healthineers) using fast spin echo (FSE) sequences and followed a consistent protocol. The scanning sequence parameters for T1WI were as follows: repetition time (TR), 400–800 ms; echo time (TE), 10–30 ms; field of view, 320 × 320 mm; matrix, 384 × 384; flip angle, 160°; number of excitations, 2; echo train length, 5; slice thickness, 3.0 mm; slice spacing, 1.0 mm. The chemical shift-selective fat saturation technique was used to accomplish fat suppression on FS-T2WI sequence. Settings for FS-T2WI were as follows: TR, 2000–4200 ms; TE, 70–110 ms; field of view, 320 × 320 mm; matrix, 320 × 320; flip angle, 150°; number of excitation, 1; echo train length, 19; slice thickness, 3.0 mm; slice spacing, 1.0 mm.

### Development of the clinical factor model

The following MRI features were collected: the maximum diameter of the tumor; shape (regular or irregular); signal (uniform or uneven); boundaries (clear or unclear); location (cervical, thoracic, lumbar, or sacrum spine).

Univariate analysis was used to compare the differences in clinical data and MRI characteristics of SSM and SPST in the training set. The significant variables in the univariate analysis were included in multiple logistic regression analysis to establish the clinical factors model. Odds ratios (ORs) as estimates of relative risk and 95% confidence intervals (CIs) were calculated for each individual factor.

### Image segmentation of tumors and radiomics feature extraction

**Fig. 2:**
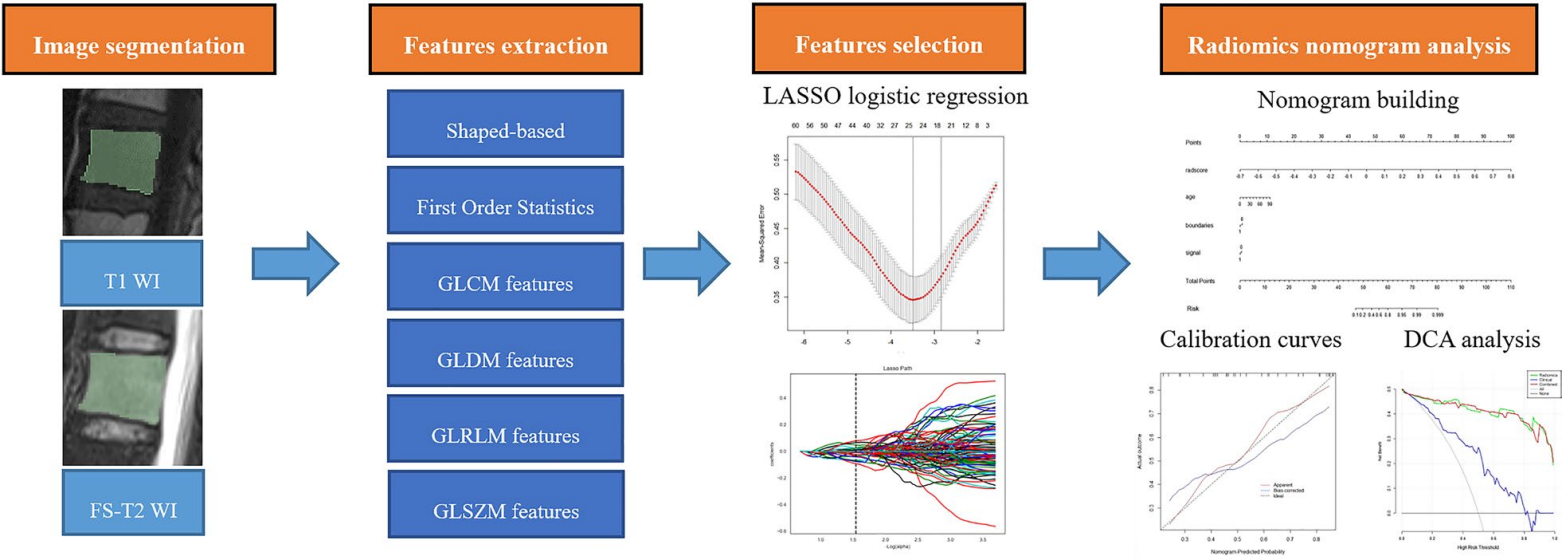
The overall workflow of the radiomics model development

The detailed radiomics workflow is shown in Fig. 2. The three-dimensional (3-D) manual segmentation of the region of interest (ROI) was performed on the RadCloud Platform (Huiying Medical Technology Co., Ltd., China). On the sagittal T1WI and FS-T2WI images, contouring was cautiously delineated within the borders of the tumors, but the adjacent normal vertebral body was not covered.. The MRI image segmentation was performed by two radiologists (reader 1, S. L.; reader 2, S. Z.) with 4 and 9 years of experience in musculoskeletal imaging, respectively.

The inter-observer and intra-observer reproducibility of feature extraction were assessed by using inter- and intra-class correlation coefficients (ICCs). Twenty MRI images (10 for SSM and 10 for SPST) were randomly selected for ROI segmentation performed by reader 1 and reader 2. Reader 1 repeated the segmentation after two weeks to evaluate the reproducibility of feature extraction. An ICC greater than 0.75 indicates good consistency of the feature extraction. Then the remaining images were segmented by reader 1.

### Construction of the radiomics signature

In order to eliminate the influence of dimension between features and make the signal intensities information consistent, the image was preprocessed before analysis in our study, which can be used to correct the intensity nonuniformity caused by the scanner's magnetic field nonuniformity during image acquisition and ensure the scale and direction were maintained when deriving the 3D features [20, 21]. Firstly, only the features with the criteria of having inter- and intra-observer ICCs greater than 0.75 were tested by one-way analysis of variance (ANOVA). Then, the Select K Best algorithm was used to obtain fit features as invariant values. And we used the least absolute shrinkage and selection operator (LASSO) regression model to identify the optimal features in the training set. Finally, the selected features were applied to build a radiomics signature. Radiomics score (Rad-score) for each patient was weighted according to their respective LASSO coefficients through a linear combination of selected features.

### Development of a radiomics nomogram model and evaluation of the performance of different models

A radiomics nomogram model was established by integrating the significant variables of clinical factors and radiomics features. The areas under the receiver operator characteristic (ROC) curve for the training and validation sets were used to quantify the diagnostic performance of the clinical factors model, the radiomics signature, and the radiomics nomogram. The Hosmer–Lemeshow test was performed to assess the goodness-of-fit of the nomogram. And we used decision curve analysis (DCA) to describe and estimate the clinical usefulness of the models by calculating the net benefits at different threshold probabilities in the whole cohort.

### Statistical analysis

All statistical analyses were conducted using SPSS software (version 25.0, IBM) and R statistical software (version 3.3.3, https://www.r-project.org). A univariate analysis was performed to test for statistically clinical and imaging differences between SSM and SPST. For categorical variables, the chi-square test or Fisher exact test was used, and the independent-samples t-test for quantitative data. One-way ANOVA was applied to compare the value of each radiomics feature for the differentiation between the two groups. the Select K Best algorithm was analyzed using the “FSinR” package [22]. The LASSO regression model was analyzed using the “glmnet” package software package [23]. The ROC curves were drawn using the “pROC” package [24]. Meanwhile, nomograms and calibration plots were generated using the “rms” package (http://CRAN.R-project.org/package=rms). The “generalhoslem” (http://CRAN.R-project.org/package=generalhoslem) and “dca.R.” (http://CRAN.R-project.org/package=DCA) packages were used to compute the Hosmer–Lemeshow test and DCA, respectively. Finally, we adopted the Delong test to estimate the AUC differences between the three models. Statistical significance was set at *p* < 0.05.

### Development of the clinical factors model

**Fig. 3:**
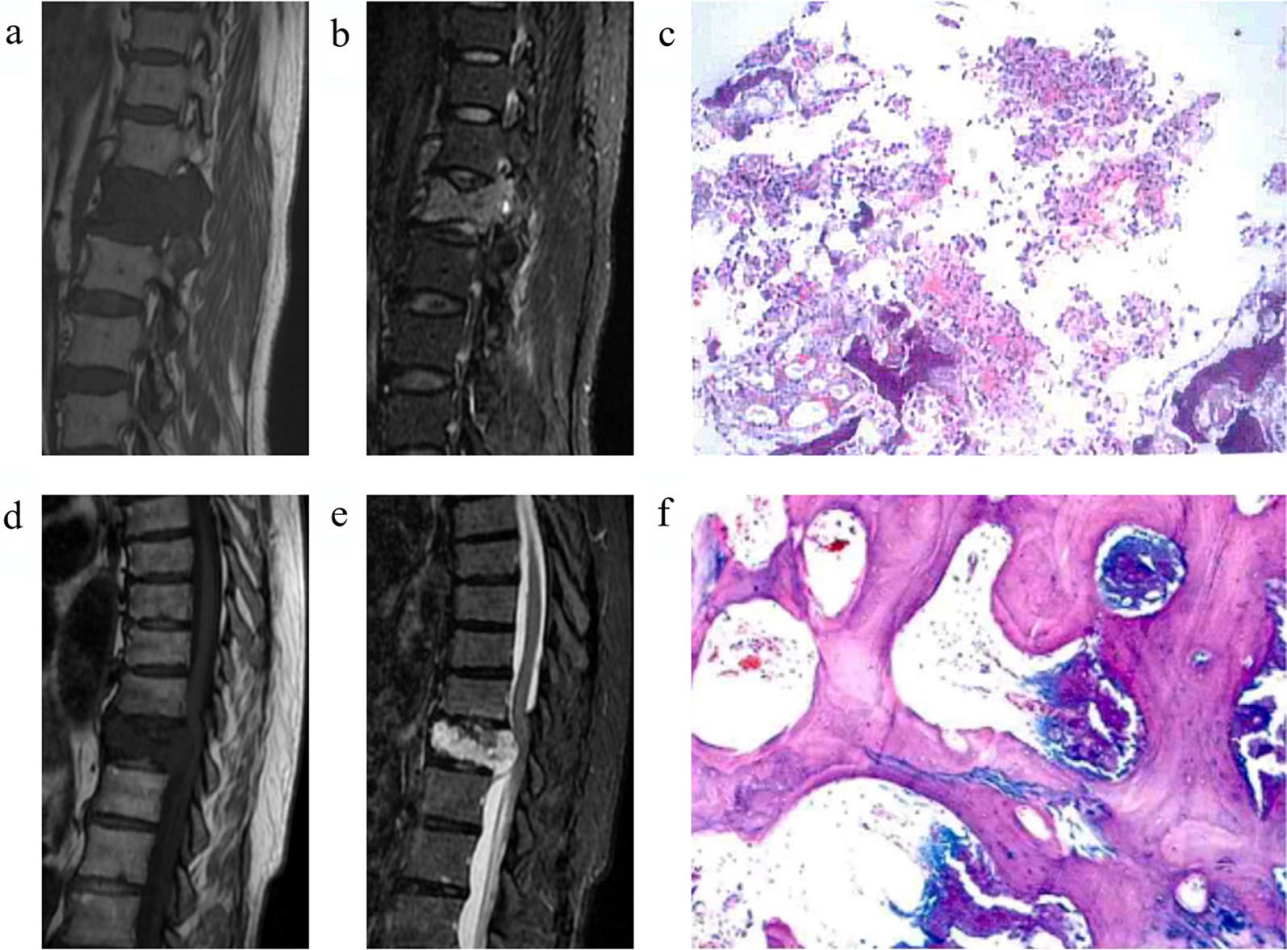
Two case examples. a, b. MR images in a 66-year-old woman with lung cancer. Sagittal T1-weighted (**a**) and sagittal FS-T2WI (**b**) show a solitary lesion at the L1 vertebral body. **c** Photomicrograph (hematoxylin and eosin staining, ×100) confirming lumbar metastatic tumor. **d**, **e** MR images in a 65-year-old woman. Sagittal T1-weighted (**e**) and sagittal FS-T2WI show a solitary lesion at the T11 vertebral body. **f** Photomicrograph (hematoxylin and eosin staining, ×400) confirming chondrosarcoma

Relevant clinical factors of the patients in the training and the validation sets are shown in Table 1. There were significant differences in age and two MRI features between SSM and SPST (Fig. 3). Multivariate logistic regression analysis revealed that age (*p* = 0.003), signal (*p* = 0.044), and boundaries (*p* = 0.002) remained as independent predictors in the clinical factors model. Tumors with older age (OR 1.060; 95% CI 1.020–1.101), a uniform signal (OR 2.582; 95% CI 1.026–6.499), or poorly defined boundaries (OR 0.209; 95% CI 0.078–0.559) were likely to be SSM.

**Table 1:** Clinical factors of the training and validation sets

Clinical factors	Training set (n = 98)			Validation set (n = 37)		
	SSM group (n = 49)	SPST group (n = 49)	*p*	SSM group (n = 20)	SPST group (n = 17)	*p*
Gender (male/female)	25/24	23/26	0.840	13/7	8/9	0.331
Age, mean ± SD, years	59.73 ± 8.41	49.16 ± 18.99	0.001	58.15 ± 11.39	51.35 ± 20.94	0.243
Maximum diameter (cm)	3.06 ± 1.38	4.87 ± 10.53	0.234	3.39 ± 1.26	4.71 ± 2.94	0.101
Shape (regular or irregular)	14/35	13/36	1.000	6/14	4/13	0.725
Signal (uniform or uneven)	30/19	18/31	0.026	12/8	7/10	0.330
Boundaries (clear or unclear)	10/39	26/23	0.001	8/12	13/4	0.045

Continuous variables are described as mean ± standard deviation, and categorical variables are presented as numbers (%)

*SSM* solitary spinal metastasis, *SPST* solitary primary spinal tumor

### Feature extraction, selection, and construction of radiomics features

**Fig. 4:**
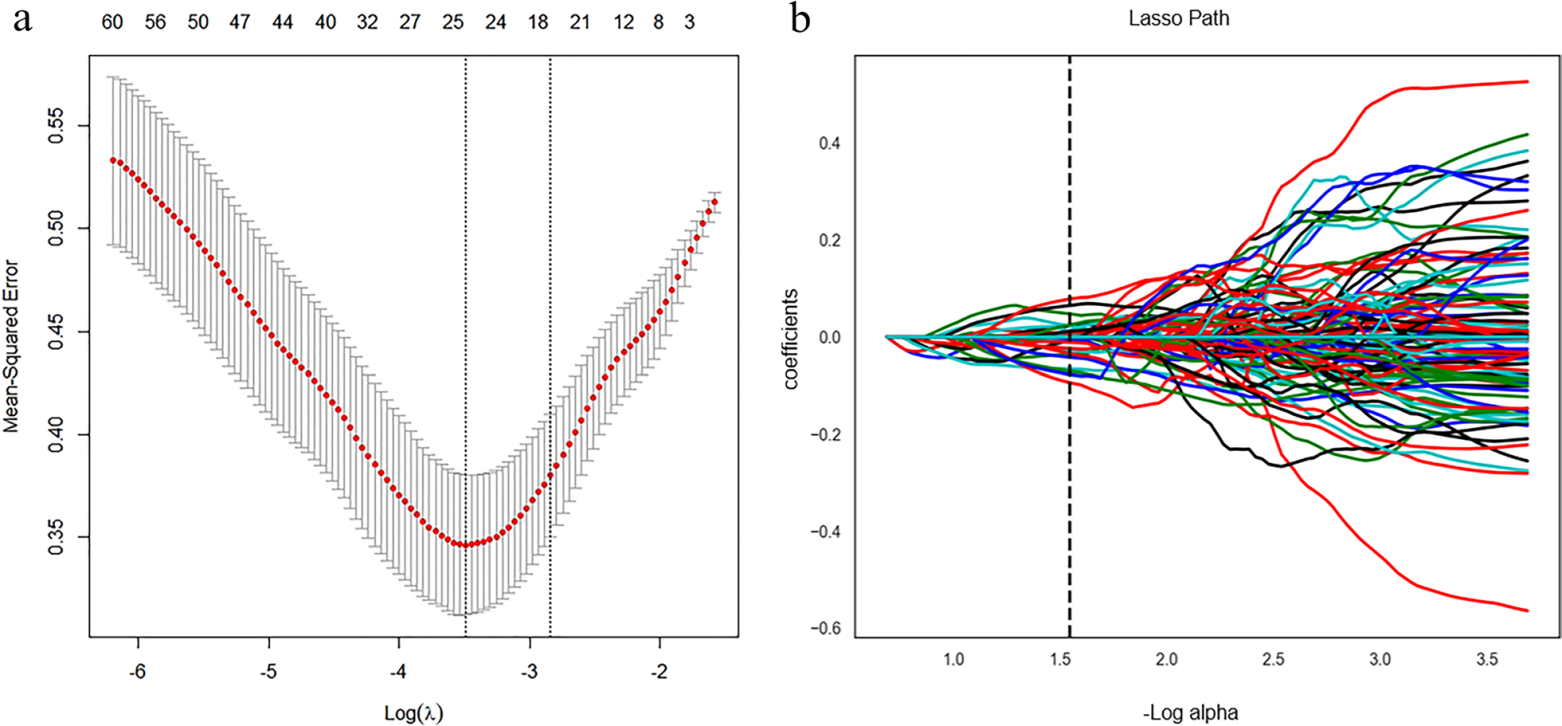
Radiomic feature selection using the least absolute shrinkage and selection operator (Lasso) cox regression model. **a** Tuning parameter (λ) selection in the Lasso logistic model was chosen using tenfold cross-validation based on the minimum criteria. The optimal values of the LASSO tuning parameter (λ) are indicated by the vertical dashed lines, and a value λ of 0.028 with log (λ) = 1.545 was chosen. **b** LASSO coefficient profiles of the 679 radiomics features. A coefficient profile plot was generated versus the selected log (λ) value using tenfold cross-validation, and 26 radiomics features were selected with non-zero coefficients

Of the 2818 radiomics features extracted from sagittal T1WI and FS-T2WI, 2732 showed a good inter-observer and intra-observer agreement with ICCs > 0.75. And 2352 radiomics features showed significant differences between SSM and SPST by one-way ANOVA. Then, 679 features obtained by the Select K Best algorithm were enrolled into the LASSO logistic regression model to select the most valuable features (Fig. 4). Finally, the radiomics signature model was built by using 26 features.

Radiomics-score = − 0.03619102 × A − 0.008243862 × B − 0.066046414 × C − 0.009470729 × D − 0.01143484 × E + 0.0071842 × F + 0.022352733 × G − 0.027389885 × H + 0.022492058 × I + 0.047207099 × J + 0.009669126 × K − 0.093820903 × L − 0.002162415 × M + 0.012730966 × N + 0.022513546 × O − 0.042216278 × P + 0.008942049 × Q + 0.024651154 × R − 0.034121703 × S − 0.070302674 × T − 0.077206832 × U + 0.064455605 × V − 0.002917858 × W + 0.011232737 × X − 0.023865389 × Y + 0.07784670 × Z.

The variables A to Z represent the selected radiomics features, which are provided in Additional file 1: Table S1. The Rad-score showed statistically significant differences between SSM and SPST (OR 6.025; 95% CI 2.522–14.523, *p* < 0.05).

### The radiomics nomogram construction and evaluation of different models

**Fig. 5:**
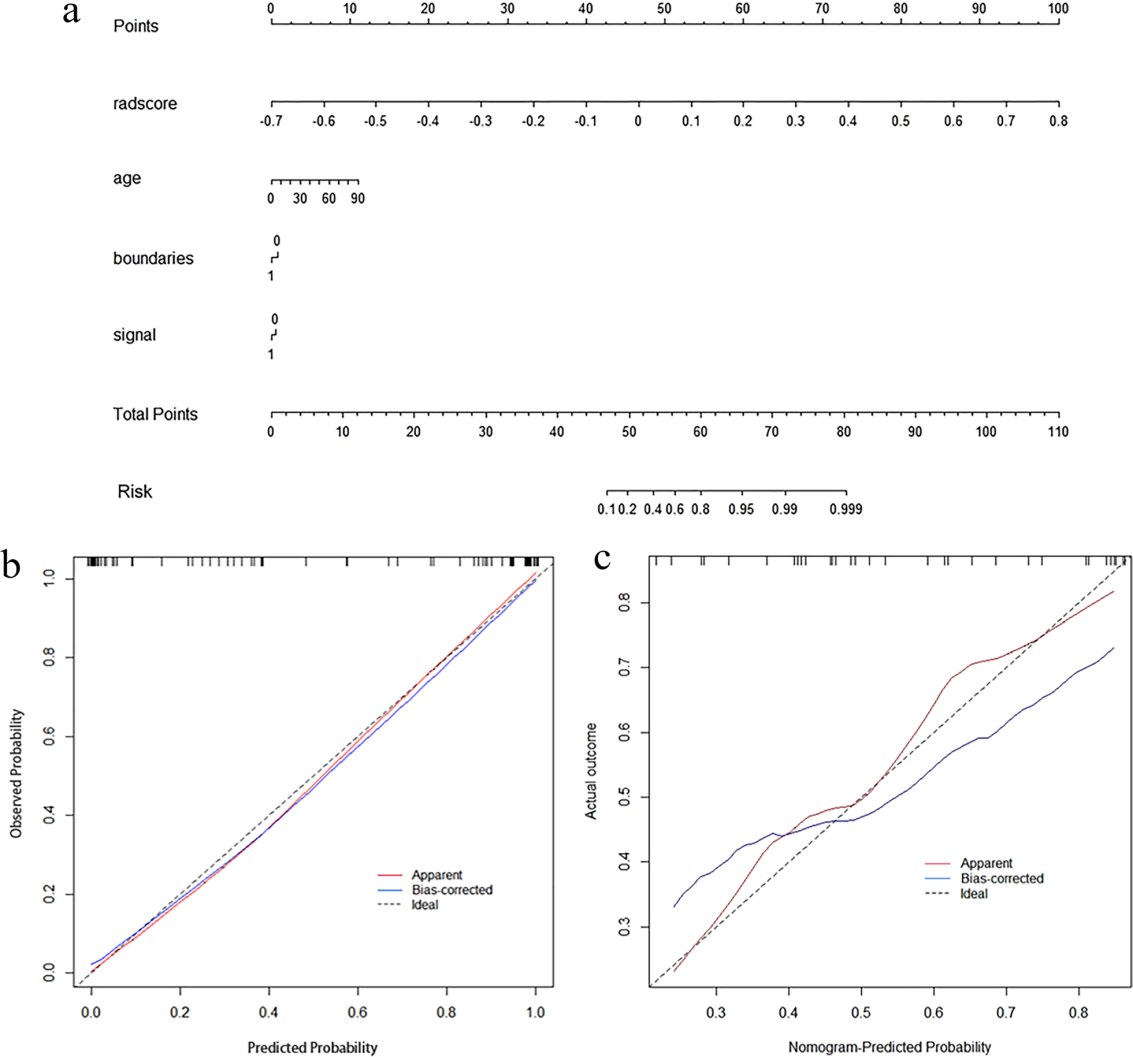
Radiomics nomogram and calibration curves. **a** Radiomics nomogram integrated with age, signal, boundaries, and radiomics signature, developed in the training set. Calibration curves of the radiomics nomogram in the training (**b**) and validation (**c**) sets. Calibration curves indicate the goodness-of-fit of the nomogram. The 45° gray line represents the ideal prediction. The apparent line is the calibration accuracy of the original date. The bias-corrected line is derived via 1000 repetitions of bootstrapping, which corrects the overfitting. A closer distance between the two curves indicates better prediction accuracy of the radiomics nomogram

The radiomics nomogram (Fig. 5a) was generated by integrating age, signal, boundaries, and radiomics signature. Figure 5b, c shows the calibration curve of the nomogram. The calibration curve demonstrated excellent calibration when applied to the validation set, accompanied by a nonsignificant Hosmer–Lemeshow test result (*p* = 0.650).

**Fig. 6:**
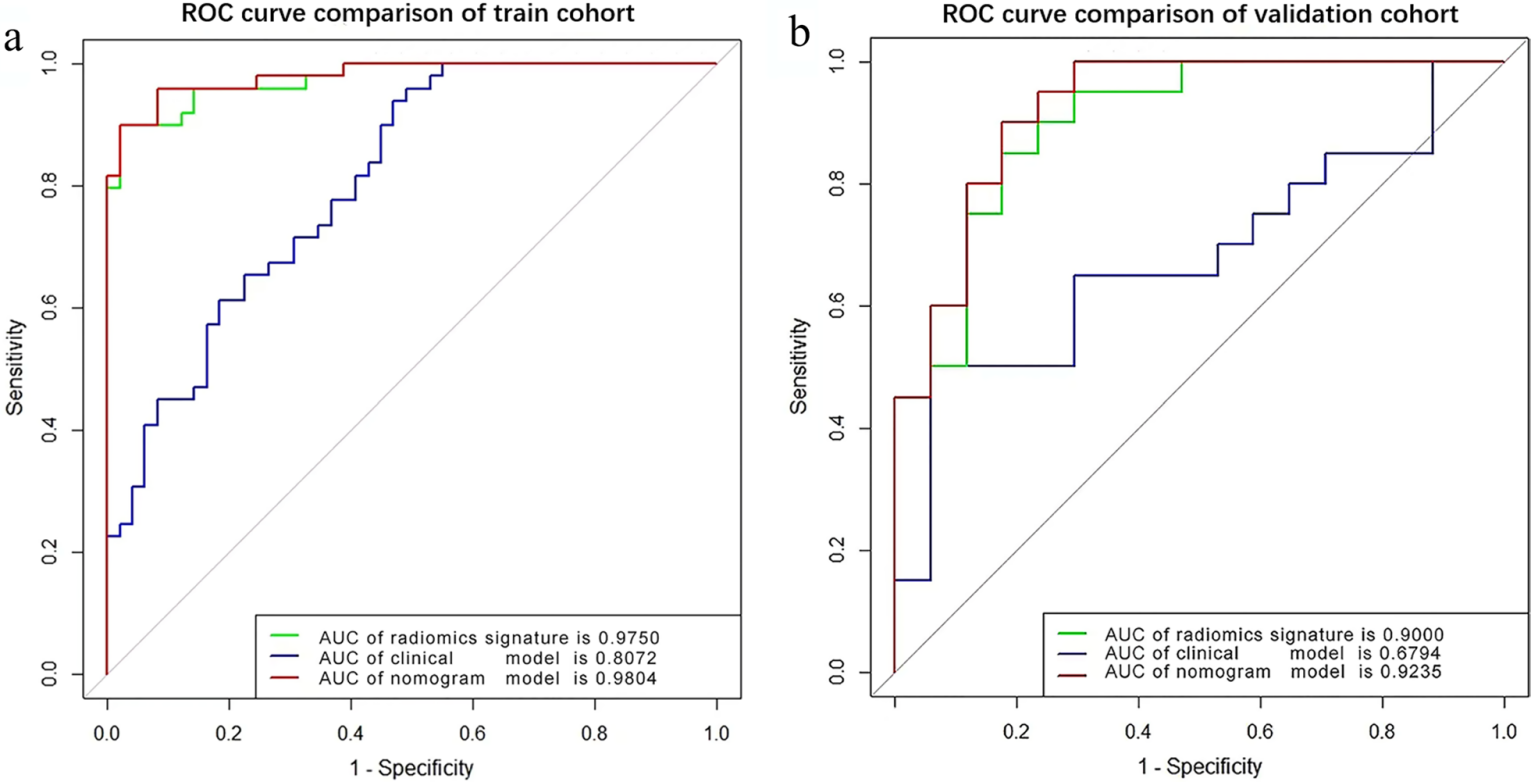
Receiver operating characteristic curves of the clinical factors model, radiomics signature, and radiomics nomogram in the training (**a**) and validation (**b**) sets, respectively

Table 2 summarizes the performance of the three models on the training and validation sets. ROC curves of the three models for both the training and validation sets are shown in Fig. 6. The radiomics nomogram model markedly outperformed the clinical factors model in both the training set (AUC 0.980 vs. 0.807, *p* < 0.01) and validation set (AUC 0.924 vs. 0.679, *p* = 0.020). However, there was no statistically significant difference between the diagnostic performance of the radiomics nomogram and that of the radiomics signature model.

**Table 2:** Diagnostic performance of the clinical factors model, the radiomics signature, and the radiomics nomogram

Set	Model	AUC (95% CI)	Sensitivity %	Specificity %	Accuracy %
Training set (n = 98)	Clinical model	0.807 (0.733–0.868)	67 (33/49)	71 (35/49)	69 (68/98)
	Radiomics signature	0.975 (0.950–0.993)	96 (47/49)	90 (44/49)	93 (91/98)
	Radiomics nomogram	0.980 (0.959–0.995)	96 (47/49)	90 (44/49)	93 (91/98)
Validation set (n = 37)	Clinical model	0.679 (0.533–0.791)	71 (12/17)	60 (12/20)	65 (24/37)
	Radiomics signature	0.900 (0.702–0.914)	88 (15/17)	75 (15/20)	81 (30/37)
	Radiomics nomogram	0.924 (0.693–0.916)	82 (14/17)	80 (16/20)	81 (30/37)

*CI* confidence interval, *AUC* area under the curve

**Fig. 7:**
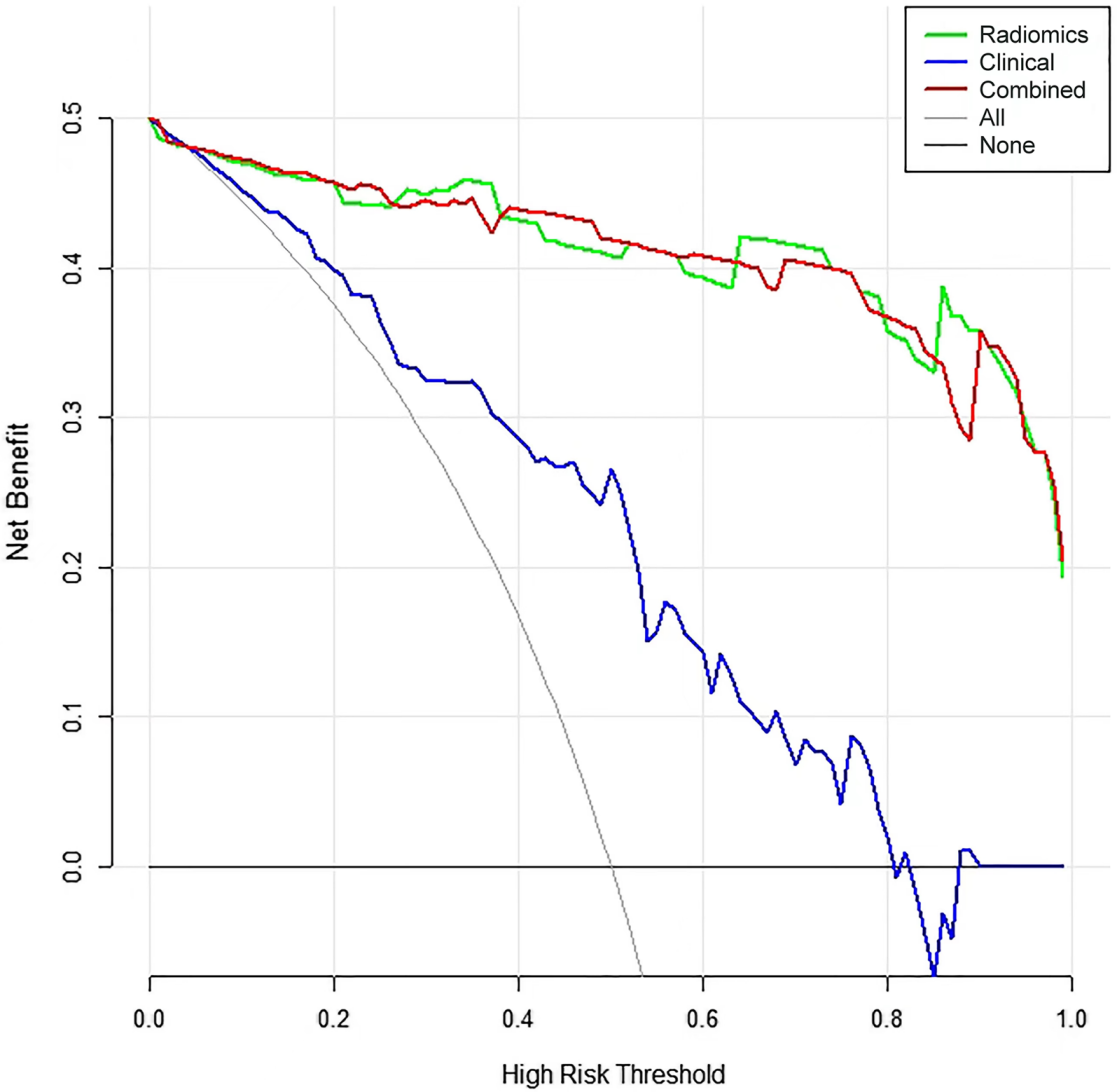
Decision curve analysis for the prediction models in the validation cohort. The y-axis measures the net benefits, while the x-axis represents the threshold probability. The red line, blue line, and green line represent the net benefit of the combined model, the clinical factor model, and the radiomics signature, respectively. "All" and "none" are two reference lines. The gray line indicates the hypothesis that all tumors are SSM. The horizontal black line indicates the hypothesis that no tumors are SSM. The DCA analysis showed that the radiomics nomogram was more beneficial than the clinical factors model

The decision curve analysis (Fig. 7) further indicated that the radiomics nomogram provided a higher net benefit and predictive ability in differentiating SSM from SPST than the clinical factors model.

## Discussion

Distinguishing SSM from SPST preoperatively is challenging and clinically important for treatment decisions and prognosis. Several reports have described radiomics as another key technique for comprehensive detection of tumor heterogeneity. It can also make up for the shortcomings of traditional imaging diagnosis [13, 14]. In situations where SSM and SPST imaging features overlap, radiomics can be a valuable additional tool. In the current study, we proposed and validated a conventional MRI-based radiomics nomogram to distinguish SSM from SPST. The nomogram model, combined with the radiomics signature, age, signal, and boundaries, achieved good performance for differentiating SSM from SPST, with AUCs of 0.980 and 0.924 in the training and validation cohorts, respectively. Compared with the clinical factors alone (training-AUC = 0.807, validation-AUC = 0.679), the nomogram model was significantly improved. It suggested that the combined nomogram model could be used as a reliable and effective method to formulate the correct treatment plan for patients with spinal tumors.

Magnetic resonance imaging with superior soft-tissue contrast and high spatial resolution plays an important role in diagnosing spinal tumor [25]. In our study, age was considered to be an independent predictor of solitary spinal metastasis. We also found that SSM usually exhibited poorly defined boundaries. The metastatic tumor shared most of the same characteristics as the primary tumor [26], and the primary tumor of metastasis in this paper had a higher degree of malignancy, which explained why the boundaries of SSM were unclear in our study. However, the clinical factors model based on age, signal, and boundaries did not achieve a high AUC (0.679 in the validation set) for differentiating SSM and SPST.

Radiomic Machine Learning and radiomics have been proposed for multiple uses in the diagnosis of spinal tumors [8, 17, 19, 27]. Sun et al. [16] developed a CT-based nomogram that combined the clinical risk factors and the radiomics signature to distinguish between benign and malignant bone tumors. And Vito Chianca et al. [8] proposed differential diagnosis of primary, malignant or metastatic tumors of the spine by radiomic machine learning. The study of Filograna et al. [28] aimed to isolate the most significant features to predict spinal metastases for oncological patients based on radiomics features, and they were more focused on the distinction between metastases and normal vertebral bodies. Another study [11] tried to identify whether the primary tumor of the metastasis was derived from the lung by radiomics and deep learning. Their findings supported the usefulness of radiomics in the diagnosis of spinal tumors. However, unlike them, we used a radiomics approach to distinguish SSM from SPST. And we considered solitary spinal metastasis harder to identify and more meaningful in the clinic.

Tumor history is important for the diagnosis of spinal metastasis [3]. But in the actual clinic, doctors are frequently unaware of the specific tumor history, so this information was not included in the clinical factors model. Even when blinded to tumor history, the radiomics signature (AUC, 0.900) and nomogram (AUC, 0.924) presented good diagnostic efficacy in this paper. Additionally, we drew the outline of lesions by 3D ROI, which covered more available sites and better reflected tumor heterogeneity than the largest cross-sectional areas [29].

However, this study had some limitations. First, this was a retrospective study, so potential selection bias was unavoidable. Second, all patients were from a single medical center, which could lead to skewed results and a lack of an external test set. A prospective multicenter study is warranted in further study. Third, another limitation was the inclusion of different types of spinal tumors in the study, which led to increased clinical heterogeneity and inevitably affected the outcome. Fourth, each ROI was manually drawn on the original image of the T1WI and FS-T2WI sequences. Thus, there is an urgent need for an automated or semiautomated ROI to reduce variability between observers.

## Conclusion

In conclusion, our study developed and validated a radiomics nomogram that combined radiomics features and clinical factors to help distinguish between SSM and SPST.

## Supplementary Information

**Additional file 1: Table S1.** Radiomics feature selection results

## Data Availability

The data sets generated and/or analyzed during the current study are available from the corresponding author on reasonable request.

## Abbreviations

3-D: Three-dimensional
ANOVA: Analysis of variance
AUC: Area under the curve
CI: Confidence interval
DCA: Decision curve analysis
FS-T2WI: Fat-saturated T2-weighted images
ICC: Inter-/intra-class correlation coefficient
LASSO: Least absolute shrinkage and selection operator
MRI: Magnetic resonance imaging
OR: Odds ratio
ROC: Receiver operator characteristic curves
ROI: Region of interest
SPST: Solitary primary spinal tumor
SSM: Solitary spinal metastasis
T1WI: T1-Weighted images
