# Supplementary material for: MRI-based radiomics nomogram for differentiation of solitary metastasis and solitary primary tumor in the spine
Source: BMC Med Imaging. 2023 Feb 9;23:29. doi: 10.1186/s12880-023-00978-8 (PMC9909949; doi:10.1186/s12880-023-00978-8)
Supplement: Supplementary file 1 — Additional file 1: Table S1. Radiomics feature selection results [file 12880_2023_978_MOESM1_ESM.docx]

**Table S1** Radiomics feature selection results

| Variables | Sequences | Types | Radiomics feature | LASSO Coefficient |
| --- | --- | --- | --- | --- |
| A | T1WI | GLCM | wavelet-LLH_glcm_ClusterShade | -0.036 |
| B | T1WI | GLDM | wavelet-LHL_gldm_LowGrayLevelEmphasis | -0.008 |
| C | T1WI | GLSZM | wavelet-HLL_glszm_ZoneEntropy | -0.066 |
| D | T1WI | GLSZM | wavelet-LHL_glszm_GrayLevelNonUniformity | -0.009 |
| E | FS-T2WI | GLSZM | wavelet-LLH_glszm_SizeZoneNonUniformity | -0.011 |
| F | T1WI | First-order | wavelet-HLL_firstorder_Minimum | 0.007 |
| G | T1WI | GLRLM | wavelet-LHL_glrlm_HighGrayLevelRunEmphasis | 0.022 |
| H | T1WI | GLSZM | wavelet-LLH_glszm_HighGrayLevelZoneEmphasis | -0.027 |
| I | T1WI | First-order | wavelet-LHL_firstorder_Mean | 0.022 |
| J | FS-T2WI | GLCM | wavelet-LLL_glcm_Imc1 | 0.047 |
| K | FS-T2WI | First-order | logarithm_firstorder_Minimum | 0.100 |
| L | T1WI | GLRLM | wavelet-LHL_glrlm_LongRunLowGrayLevelEmphasis | -0.094 |
| M | FS-T2WI | First-order | squareroot_firstorder_Variance | -0.002 |
| N | FS-T2WI | First-order | squareroot_firstorder_Minimum | 0.013 |
| O | FS-T2WI | First-order | logarithm_firstorder_Skewness | 0.023 |
| P | FS-T2WI | GLSZM | wavelet-LHH_glszm_GrayLevelNonUniformityNormalized | -0.042 |
| Q | T1WI | First-order | squareroot_firstorder_Skewness | 0.009 |
| R | FS-T2WI | First-order | squareroot_firstorder_Skewness | 0.025 |
| S | FS-T2WI | GLCM | wavelet-LLL_glcm_Imc2 | -0.034 |
| T | T1WI | First-order | logarithm_firstorder_Kurtosis | -0.070 |
| U | T1WI | First-order | wavelet-HLL_firstorder_RootMeanSquared | -0.077 |
| V | FS-T2WI | First-order | lbp-2D_firstorder_10Percentile | 0.064 |
| W | T1WI | GLSZM | wavelet-HHL_glszm_SmallAreaHighGrayLevelEmphasis | -0.003 |
| X | FS-T2WI | First-order | wavelet-HLL_firstorder_Mean | 0.011 |
| Y | FS-T2WI | First-order | wavelet-LHL_firstorder_Skewness | -0.024 |
| Z | FS-T2WI | GLCM | wavelet-LHL_glcm_ClusterProminence | 0.078 |

T1WI, T1-weighted images; FS-T2WI, fat-saturated T2-weighted images; GLCM, gray-level co-occurrence matrix; GLDM, gray-level dependence matrix; GLRLM, gray-level run length matrix; GLSZM, gray-level size zone matrix.
